# Perspectives on competency-based feedback for training non-specialists to deliver psychological interventions: multi-site qualitative study of the EQUIP competency-based approach

**DOI:** 10.1192/bjo.2024.37

**Published:** 2024-06-03

**Authors:** Abdelrhman Elnasseh, Varun S. Mehta, Gergana Manolova, Gloria A. Pedersen, Shannon Golden, Liyam Eloul, Frezgi Gebrekristos, Pamela Y. Collins, Teresia Mutavi, Anne W. Mbwayo, Muthoni Mathai, Tessa Concepcion, Rozane El Masri, Frederik Steen, Jerome T. Galea, Carmen Contreras, Josephine Akellot, Rosco Kasujja, Samuel Wasereka, Byamah Brian Mutamba, Wietse A. Tol, Mansurat Raji, Sacha Moufarrej, Alison Schafer, Brandon A. Kohrt

**Affiliations:** Center for Global Mental Health Equity, Department of Psychiatry and Behavioral Health, The George Washington School of Medicine and Health Sciences, USA; London School of Hygiene and Tropical Medicine, UK; The Center for Victims of Torture, Addis Ababa, Ethiopia; Johns Hopkins Bloomberg School of Public Health, Johns Hopkins University, USA; Department of Psychiatry, School of Medicine, College of Health Sciences, University of Nairobi, Kenya; Department of Global Health, University of Washington, USA; Research and Development Department, War Child, Beirut, Lebanon; Research and Development Department, War Child, Amsterdam, The Netherlands; School of Social Work, University of South Florida, USA; Socios En Salud, San Isidro, Peru; HealthRight International, Kampala, Uganda; Department of Mental Health, Makerere University, Uganda; Butabika Hospital, Kampala, Uganda; Section of Global Health, Department of Public Health, University of Copenhagen, Denmark; Department of Mental Health and Substance Abuse, World Health Organization, Geneva, Switzerland; Department of Psychiatry & Behavioral Sciences, Duke University School of Medicine, USA

**Keywords:** Education and training, transcultural psychiatry, qualitative research, psychosocial interventions, low- and middle-income countries

## Abstract

**Background:**

The use of feedback to address gaps and reinforce skills is a key component of successful competency-based mental health and psychosocial support intervention training approaches. Competency-based feedback during training and supervision for personnel delivering psychological interventions is vital for safe and effective care.

**Aims:**

For non-specialists trained in low-resource settings, there is a lack of standardised feedback systems. This study explores perspectives on competency-based feedback, using structured role-plays that are featured on the Ensuring Quality in Psychosocial and Mental Health Care (EQUIP) platform developed by the World Health Organization and United Nations Children’s Fund.

**Method:**

Qualitative data were collected from supervisors, trainers and trainees from multiple EQUIP training sites (Ethiopia, Kenya, Lebanon, Peru and Uganda), from 18 key informant interviews and five focus group discussions (*N* = 41 participants). Qualitative analysis was conducted in Dedoose, using a codebook with deductively and inductively developed themes.

**Results:**

Four main themes demonstrated how a competency-based structure enhanced the feedback process: (a) competency-based feedback was personalised and goal-specific, (b) competency-based feedback supported a feedback loop, (c) competency-based feedback supported a comfortable and objective feedback environment, and (d) competency-based feedback created greater opportunities for flexibility in training and supervision.

**Conclusions:**

A better understanding of the role of feedback supports the implementation of competency-based training that is systematic and effective for trainers and supervisors, which ultimately benefits the learning process for trainees.

## Need for quality in mental health education

In June 2022, the World Health Organization (WHO) released the ‘World Mental Health Report: Transforming Mental Health For All’, concluding with three main goals, one of which is strengthening mental health systems.^1^ The report calls for building competencies for mental healthcare. The WHO and United Nations Children’s Fund (UNICEF) developed Ensuring Quality in Psychosocial and Mental Health Care (EQUIP). EQUIP (www.equipcompetency.org) is a freely available resource with standardised competency assessment tools to implement competency-based education in training and supervision.^2^ However, to date, the feasibility and acceptability of competency-based education when training non-specialists have not been evaluated.

## Competency-based education

Competency-based education refers to teaching strategies that use an identified list of skills that trainees must demonstrate for successful completion of training. In competency-based education, the progress of demonstrable skills is tracked over time, and training is adjusted at the group and individual levels based on achieving predetermined milestones.^3^ Competency-based education was introduced by behavioural psychologists in the 1940s, with a revival in the 1990s resulting in increased use in multiple educational spheres, including healthcare education such as through the Accreditation Council for Graduate Medical Education.^4^ In 2010, a consensus definition of competency-based education was proposed in the healthcare field: competency-based education is ‘an approach to preparing physicians for practice that is fundamentally oriented to graduate outcome abilities and organized around competencies derived from an analysis of societal and patient needs. It de-emphasizes time-based training and promises greater accountability, flexibility, and learner-centeredness’.^5^ Competency-based education initiatives are gaining recognition worldwide, with leading medical schools in sub-Saharan Africa, such as Makerere University, adopting the practice.^6^

## Feedback as a critical component of competency-based training

Feedback is a hallmark of successful competency-based education interventions because it continuously informs students and trainees on how to better achieve competencies, resulting in tailored educational support.^3,7,8^ For example, if a student or trainee is not achieving competency A but is achieving competency B, the feedback and associated educational time and effort can be targeted toward improving competency A.^3,9^ Competency-based feedback involves iteratively evaluating skills displayed by trainees through role-plays or other observations.

A further technique to enhance competency-based feedback is the feedback loop, wherein students or trainees respond with their feelings about the feedback. This can support agreement on how trainees attempt to improve their performance. Based on these efforts, teachers or trainers check in again on competency performance, creating a loop that reinforces trainees’ skills until competencies are attained.^9–12^ Feedback loops are useful because they continually give learners points to work toward. This creates a sense of continued growth in specific skills rather than perceptions of stagnation associated with knowledge-based approaches that do not incorporate continuous feedback.^13,14^

Competency-based education may have received limited attention in global health training because it is not as well-studied in short courses such as those commonly used in global mental health, which typically last around 2 weeks.^6^ Competency-based education has historically been proposed as a years-long education strategy, but the EQUIP platform provides guidance on incorporating competency-based education in brief trainings of a few days to a few weeks, followed by supervision, which may last a few months.^6^ Moreover, these brief trainings are typically of fixed duration (e.g. 2-day training, 10-day training) on a particular intervention. Therefore, it may be seen as more difficult to have a competency-based approach in brief training compared with the flexibility built into a multi-year health professional training programme. To date, most evaluations of trainings of non-specialists in manualised interventions are usually through attendance, written knowledge tests or satisfaction surveys (e.g. ‘Did you feel the training covered all the necessary skills you need for your work?’).^15^ Therefore, one of the objectives during the development of EQUIP was to evaluate the feasibility and acceptability of using competency-based approaches in existing curricula for non-specialists working with a range of manualised interventions in different settings around the world. This current analysis uses qualitative data to understand the potential for competency-based feedback using EQUIP and, generally, in the global mental health field to improve mental health task-sharing training and supervision.

## Method

### EQUIP pilot implementation

During the development of the EQUIP platform, a multi-site qualitative study was conducted from 2018 to 2020 to explore if and how a competency-based approach benefitted training and supervision outcomes when preparing non-specialists to deliver psychological interventions. EQUIP is a platform that guides trainers and supervisors in designing and amending training and supervision, using a competency-based approach.^16^ EQUIP functions as a cross-intervention resource for training and supervising non-specialists in delivering various mental health interventions and basic psychosocial support competencies.

EQUIP includes a series of competency assessment tools for psychological, psychosocial and mental health interventions. Competency assessments can be used in structured role-plays, observing mental health sessions (live or recorded) and self- or peer-ratings during training or supervision. Competencies are rated by checking off observed behaviours (helpful or potentially unhelpful/harmful), and then rating each competency on 4 levels: level 1, ‘any unhelpful or harmful behaviour’; level 2, ‘no harmful behaviours, but not all basic skills’; level 3, ‘no harmful behaviours and all basic skills’; and level 4, ‘no harmful behaviours, all basic skills and at least one advanced skill’. All competency assessment tools on the platform use this structure. The foundational tool on the platform is Enhancing Assessment of Common Therapeutic Factors (ENACT).^17,18^ ENACT has 15 items to assess common therapeutic factors (e.g. verbal and non-verbal communication skills, empathy, collaboration and promoting hope).

During the development phase, EQUIP was tested in multiple settings with different mental health interventions, including the Thinking Healthy Program (THP) in Peru, Early Adolescent Skills for Emotions (EASE) in Lebanon, Problem Management Plus (PM+) in Ethiopia, Group Interpersonal Psychotherapy (Group IPT) in Uganda, Trauma-Focused Cognitive Behavioral Therapy (TF-CBT) in Kenya, and Common Elements Treatment Approach (CETA) in Zambia.^19–23^ The current results are drawn from qualitative interviews conducted in five sites where an EQUIP competency-based training or supervision approach was used and in-depth qualitative data were collected (Peru, Ethiopia, Kenya, Lebanon and Uganda).

In the context of this study, training and research teams in each site contributed to developing the EQUIP resources, including revising and adapting the competency assessment tools and designing structured role-plays. To pilot test the feasibility and acceptability, structured role-plays were conducted before the training of non-specialists. The pre-training role-plays focused on the foundational helping skills with ENACT, and the post-training role-plays also included assessment of ENACT as well as optional treatment-specific competency assessment tools.

The competency-based feedback came about by reviewing pre-training role-play assessment results and tailoring training components to areas of strength and weakness. Group and individual feedback during the training was based on competency assessment results. Then, the competency assessment role-play after the training was used to give feedback and tailor the supervision according to strengths and weaknesses. In training and supervision, feedback was structured based on competency-based assessment tools, with a learning module on competency-based feedback available on the EQUIP platform. A detailed description of the competency-based training approach has been published from the implementation in Lebanon.^24^

### Qualitative study participants

The partners (see Table 2) for the EQUIP pilot were chosen through a competitive process based on both a history of demonstrated implementation of psychological interventions and expertise in qualitative and quantitative research. As part of the selection process, local researchers with linguistic and cultural expertise were required. Existing research staff with a prior history in conducting qualitative interviews collected the data for this project. Qualitative methods were used to evaluate the feasibility and acceptability of using competency-based feedback in these brief trainings and subsequent supervision. In each site of this substudy, trainers and supervisors using EQUIP resources were interviewed at training sites whenever possible. For trainees, a convenience sample was approached based on their availability to participate in qualitative interviews.

Two qualitative methods were used: key informant interviews (KIIs) and focus group discussions (FGDs). The interview guides were developed by the EQUIP research leadership team in consultation with each collaborating organisation. The guides used a semi-structured format, wherein theme-based, open-ended questions were followed by various prompting to support the flow of the discussion. Themes in the qualitative interview included assessing the usefulness of competency-based assessments in role-plays; in-person versus remote training; scaling up of EQUIP; and delivery of training, feedback and supervision.

All procedures contributing to this work comply with the ethical standards of the relevant national and institutional committees on human experimentation and with the Helsinki Declaration of 1975, as revised in 2008. All procedures involving human patients received ethical approval to conduct the study, provided by the WHO (ERC.0003437), George Washington University Institutional Review Board (NCR191797), Tigray Health Research Institute, Kenyatta National Hospital-University of Nairobi Ethics and Research Committee, University of Washington Institutional Review Board, St. Joseph University, Beirut, Lebanon, Comité Institucional de Ética en Investigación Institutional Review Board, Dirección de Redes Integradas Lima Nortes, Cayetano Heredia University Institutional Review Board and Milmay Uganda Research and Ethics Committee. Written informed consent was obtained from all participants. The trial was registered with Clinicaltrials.gov (identifier NCT04704362).

### Qualitative data analysis

The overall EQUIP qualitative process was a distributive and collaborative process, with aspects completed by local research teams and other aspects completed by the core EQUIP team. For example, local research partners were involved in developing the codebook and reviewing code summaries for face validity in their setting, while the core EQUIP team were involved in coding the data and writing code summaries.

Inductive and deductive coding techniques were used to create the codebook to code FGDs and KIIs using Dedoose version 9.0.17 for Windows (SocioCultural Research Consultants, California, USA; see www.dedoose.com). Four researchers coded the data, and interrater reliability of 0.7 or greater among coders was established, indicating agreement. The team coded 23 interview transcripts, and ten key codes were identified. Once all qualitative materials were coded, code queries were generated for the feedback and supervision codes. In addition to selecting the feedback code, the team used the supervision code, given that supervision helped consolidate feedback to trainees and that the competency-based feedback approach helped inform the supervision process. These code queries were then charted into tables to compare various stakeholders’ experiences. Then, relevant themes and subthemes evident in the data were identified. Cross-country findings on each theme were summarised for KIIs and FGDs with supervisors, trainers and trainees who participated in various psychological intervention trainings specific to each site (e.g. Ethiopia using PM+, Peru using THP), with a particular focus on their experiences with giving and receiving feedback structured around competency-based assessments during training and supervision. Uncertainties in coding and themes were discussed with the research coding team and resolved by consensus. The current analysis focuses on the feedback and supervision codes. Future qualitative studies will report on outcomes of other components of the EQUIP model mentioned above, such as role-plays, scaling up of EQUIP and training delivery. We have included full details on the qualitative data collection and analysis using the Consolidated Criteria for Reporting Qualitative Studies (COREQ) criteria (see Supplementary File 1 available at https://doi.org/10.1192/bjo.2024.37).^25^

## Results

Four participating sites (Ethiopia, Lebanon, Peru, Uganda) conducted KIIs (18 total) with trainers, supervisors or trainees. FGDs, separated into groups of either trainers and supervisors only or trainees only, were conducted in Lebanon (two FGDs, *n* = 12 participants) and Kenya (three FGDs, *n* = 11 participants), resulting in 41 total participants. Fifteen participants were trainers or supervisors, and 26 were trainees (Tables 1 and 2).

**Table 1:** Trainer/supervisors and trainees participating in qualitative interviews

		Types of participants		Qualitative method	
	Total	Trainers/		Key informant	Focus group
Country	participants	supervisors	Trainees	interviews	discussions
Ethiopia	4	4	0	4	0
Kenya	11	5	6	0	3 (*n* = 11)
Lebanon	13	1	12	1	2 (*n* = 12)
Peru	9	5	4	9	0
Uganda	4	0	4	4	0
Total	41	15	26	18	5 (*n* = 23)

**Table 2:** Background of trainers/supervisors and trainees

Country (site)	Psychological intervention training	Trainers and supervisors	Trainees
Ethiopia	Problem Management Plus (PM+)	Experienced MHPSS trainer/supervisors and psychosocial counsellors employed by the Center for Victims of Torture	Non-specialists in mental healthcare trained in a 10-day PM+ training with a 3-day refresher
Kenya	Trauma-Focused Cognitive Behavioral Therapy (TF-CBT)	Experienced trainers/supervisors in TF-CBT	Non-specialists in mental healthcare trained in a 10-day TF-CBT based training in a brief MHPSS intervention and ongoing supervision in this therapy
Lebanon	Early Adolescent Skills for Emotions (EASE)	Experienced trainer/supervisor, trained in EASE by EASE master trainer within War Child Holland. Trained in how to adjust the training into a competency-driven EASE training	Non-specialists in mental healthcare trained in an 8-day EASE training
Peru	Thinking Healthy Program (THP)	Trainers/supervisors employed by Socios En Salud	Non-specialists in mental healthcare trained in a 10-day THP training
Uganda	Group Interpersonal Psychotherapy (Group IPT)	Experienced trainer/supervisors in Group IPT and experienced in working with Village Health Teams (community health workers) trained by HealthRight International	Village Health Teams trained in a 7- to 14-day Group IPT training

MHPSS, mental health and psychosocial support.

**Fig. 1:**
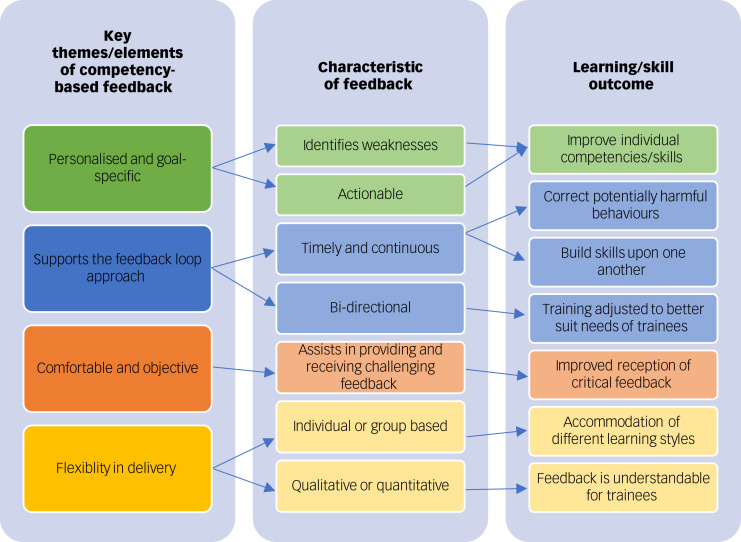
Key themes and outcomes of competency-based feedback.

Supervisors, trainers and trainees discussed various elements of feedback that contributed to trainee learning and skill attainment. From our data, we identified four major themes, which appeared as follows: (a) competency-based feedback was personalised and goal-specific; (b) competency-based feedback supported a feedback loop; (c) competency-based feedback supported a comfortable and objective feedback environment; and (d) competency-based feedback created greater opportunities for flexibility in training and supervision. Themes that represent the feedback experience of participants are further described below with supporting excerpts from KIIs and FGDs (see Fig. 1).

### Theme 1: Competency-based feedback was personalised and goal-specific

Participants in four of the five study countries (Kenya, Lebanon, Peru and Uganda; six out of 18 KIIs and four out of five FGDs) endorsed that using competency-based assessment tools to structure feedback helped to give personalised and goal-specific feedback.

#### Subtheme 1.1: Identifying areas of growth

Trainees noted having more personalised feedback helped them identify weaknesses and understand which skills they needed to improve. During training, trainees received specified feedback for each competency, such as verbal and nonverbal cues. Additionally, trainers noted that trainees learned at varying paces and displayed different areas of growth, so trainers gave personalised feedback to account for differences between trainees.

‘They [trainers] told me I had to improve rapport, self-harm assessment and keep practicing. It [feedback] seemed good to me that they are always aware of me, how I was achieving it [competencies], or how I was developing.’ (Peru, KII, trainee, #T2)

‘The area I was weak in was I wasn't assuring the client of confidentiality in every session. I also lacked assessing self-harm … I was tending to ignore it because, for me, it looked like a repetition.’ (Kenya, FGD, trainee, #T1)

#### Subtheme 1.2: Actionable feedback

Goal-specific feedback gives trainees actionable steps to improve skills. Trainees reported that their feedback specifically had examples of skill improvement rather than merely stating positive comments and criticisms. While providing feedback, trainers and supervisors had a guide to structure more actionable feedback. For example, some reported re-enacting scenarios or using role-plays to demonstrate how to help trainees improve weaknesses.

‘You ask [trainees] what will make the client uncomfortable … and they will tell you that maybe [the trainees] are blaming [clients] for what happened, and maybe [the trainees] are not assuring [the clients] of confidentiality.’ (Kenya, FGD, trainer, #T1)

### Theme 2: Competency-based feedback supported a feedback loop

Participants across all five country sites, including trainers and trainees, noted that feedback was timely, continuous, and bidirectional (four KIIs, one FGD). The feedback-loop approach was used throughout the training. In addition to providing timely and continuous feedback, feedback loops use trainees’ feedback to adjust future trainings.

#### Subtheme 2.1: Timely and continuous feedback

Structuring feedback on competencies aided the feedback-loop approach. With structured feedback, trainers reported being able to pinpoint which competency is weaker, and thus give feedback in a timelier manner. In addition, when a weaker competency was identified, trainers gave continuous feedback on that competency until improvement was observed in subsequent assessments. Trainers noted that prompt feedback allowed trainees to incorporate feedback into the following training sessions. Furthermore, the timeliness of feedback enabled trainees to catch mistakes early rather than having to unlearn ineffective behaviours.

‘100% like [participant 2] was saying, the feedback was directly after we finished; for instance, if we did not know how to deal with the situation, Trainer #03 used to tell us you were supposed to do this and that, or you were supposed to do this. So, done like this, it sticks in our minds directly.’ (Lebanon, FGD, trainee, #P1)

‘It was like a build-up, like they [our skills] were improving a step further any time the training was done, then [our] skills enhanced during supervision, and then our skills got even better during the delivery.’ (Kenya, FGD, trainer, #T1)

#### Subtheme 2.2: Bidirectional feedback

In addition to adjusting training based on trainees’ competency-based assessment results, participants reported benefits from having bidirectional feedback. For example, trainees wanted to receive feedback on assessing suicide and ensuring confidentiality, as these competencies were commonly perceived as difficult. Also, trainees requested additional training on skills not presented in the training, such as how to aid distressed couples. Trainees also provided feedback to trainers or supervisors, such as commenting on the difficulty of training or the perceived fairness of feedback.

‘[We] asked how [trainees] felt about the feedback [the trainers] gave them.’ (Peru, KII, trainer/supervisor, #T2)

‘It would be nice if [trainers] would make training farther from our village … This will help us concentrate and give attention for our training so that we would grasp adequate knowledge … [Trainees] have mentioned that the competency-based skill assessment is a little bit difficult because they did not have experience in counselling.’ (Ethiopia, KII, trainer/supervisor, #12)

### Theme 3: Competency-based feedback supported a comfortable and objective feedback environment

Participants in four sites endorsed that using competency-based assessment tools to structure feedback helped manage instances of giving and receiving challenging feedback (Kenya, Lebanon, Peru and Uganda; six KIIs, three FGDs). Supervisors and trainers expressed that challenging feedback was necessary to help correct harmful behaviour displayed by trainees.

#### Subtheme 3.1: Helped in giving and receiving challenging feedback

Some trainees reported feeling anxious about receiving feedback if they received a low score on the ENACT assessment tool, and some trainers reported difficulty addressing lower scores, such as when a trainee displayed harmful behaviour. For instance, the trainer would struggle with how to discuss with a trainee that they were showing judgement, interrupting or using inappropriate language with a pretend client during the role-play. Similarly, some competencies were more challenging to give and receive feedback on than others. For example, trainers found it easier to provide feedback on normalising behaviour versus ensuring confidentiality. Overall, when feedback was structured on competency-based tools, most trainees reported feeling receptive to feedback because it felt ‘objective’ and was delivered with a well-explained rationale. Trainers or supervisors noted that feedback was ‘comfortable to give’.

‘It was comfortable on my side unless … maybe about confidentiality or maybe someone has not done rapport building well. I discuss the potentially harmful behaviours … I discuss it in a way that I am training them … then go through the role-play.’ (Kenya, FGD, supervisor, #S2)

‘Let's say I was role-playing somebody who was sad, then the [trainee] will say don't be sad … you shouldn't cry etc. … I don't know if we can call this harmful, but it is definitely not helpful, right? Because it is not validating the emotions … so, now I would address it directly … I think because lots of the time, people do harmful things without really realising.’ (Lebanon, KII, trainer, #T3)

‘[The trainer] was giving feedback, and I was seeing it as good, objective and accurate.’ (Lebanon, FGD, trainee, #P3)

### Theme 4: competency-based feedback created greater opportunities for flexibility in training and supervision

Participants, including trainers and trainees in four countries, endorsed that using competency-based assessment tools to structure feedback allowed for multiple methods for delivering feedback (Kenya, Peru, Lebanon and Uganda; four KIIs, three FGDs).

#### Subtheme 4.1: Feedback in groups or individually

Given that feedback was based on a structured competency tool, trainers or supervisors had the flexibility to deliver feedback based on their preferences and constraints, while also accommodating trainees’ different learning styles. For example, trainers or supervisors gave individual feedback on competencies that needed improvement or formed groups based on common weaknesses. Individually, trainers called, met with or emailed trainees regarding their personalised feedback. Some trainees preferred group feedback because they felt less singled out, whereas others mentioned that individual feedback was more beneficial because it felt more personalised.

‘It [feedback] was presented individually … they gave us the feedback by talking to us, telling us that what you [trainees] have done and this part needs to be corrected.’ (Uganda, KII, trainee, #24)

‘For the training, it [feedback] was in a group way based on the results obtained on the [EQUIP] platform, and for the supervision, the communication [feedback] was personalised based on the results of the post-training evaluation and also the development of the sessions.’ (Peru, KII, trainer/supervisor)

#### Subtheme 4.2: Feedback delivered qualitatively or quantitively

Feedback based on competencies was given as numerical scores and verbal or written commentary. Although some trainees mentioned receiving scores were useful, most found greater benefit in written or verbal feedback. Numerical scores gave trainees an easy and objective way to understand their progress, whereas verbally communicated feedback gave them more detailed, comprehensive feedback.

‘[After receiving numerical feedback] I was able to see what are my strong and my negative points … but I also would like that the person who assessed me that they also write me notes [qualitative feedback] as actions for me, not only numbers, so that I improve them. So, I would like to know exactly where the mistakes were like you said this word, you did this action, you made this look.’ (Lebanon, FGD, trainee, #P3)

‘Receiving the scores on the pre-assessments was useful more than anything.’ (Lebanon, KII, trainer, #T3)

### Challenges expressed about feedback

Participants reported various challenges in giving and receiving feedback. Because of schedule restraints, some trainees expressed not receiving feedback as soon as they would have liked to, and trainers reported difficulty finding the time to give both individual and group feedback. Trainees mentioned that receiving feedback on multiple competencies at once can be overwhelming. Additionally, after receiving feedback, some trainees felt pressured to ‘be perfect’. Trainers, at times, were afraid to give feedback because they might deliver in a way that would upset the trainees.

## Discussion

Feedback in competency-based training focuses on ensuring vital skills for developing trainees. In line with existing literature that identifies advantages of competency-based feedback for improving learning outcomes,^26–28^ our qualitative findings describe the response to integrating competency-based feedback into training non-specialists to provide psychological interventions. Participants suggested that competency-based feedback loops ensured continuously modifiable training. Furthermore, competency-based feedback combined elements of effective feedback into an integrated approach that enhanced the training process.

Trainees or students should receive feedback appropriate to their current level of learning.^29^ Therefore, personalised and goal-specific feedback provided trainees with a map of where they are in the learning process, including their strengths and weaknesses, expectations for their current level of competence and steps needed to accomplish their goals. Trainees noted that structuring feedback based on competencies allowed trainers to deliver specific feedback more effectively, providing trainees with clearer goals and ultimately improving their skills to deliver care.

Delaying feedback can hinder reinforcing learning and correcting poor performance.^30,31^ A feedback-loop approach has the elements of timely, continuous and bidirectional feedback. Using competencies to structure feedback helped identify trainees’ weaknesses, which enhanced a feedback-loop approach. By effectively identifying trainees’ weaknesses, trainers and supervisors gave feedback more readily and continuously reinforced positive behaviours and corrected harmful ones. Also, when trainees could better identify their weaknesses, they were able to provide feedback to trainers and supervisors to help tailor future trainings.

Kluger and DeNisi^32^ conducted a meta-analysis of feedback interventions, including 131 studies and 12 652 participants, demonstrating that up to a third of feedback interventions could adversely affect performance. Some adverse effects were a result of feedback being perceived as challenging and focusing on employees’ mistakes rather than accomplishments.^32,33^ Critical feedback can potentially impede the learning process if not provided appropriately. However, this should not lead trainers to shy away from such feedback, because honest, critical feedback is key to the effectiveness of the process.^34^ Competency-based feedback mitigated the potentially sensitive nature of challenging feedback by focusing it on specific skills. This structure reduced the possibility of subjective feedback and the perception of feedback as a slight on the trainee's character.

There are various perspectives regarding the most efficacious method to deliver feedback. Some studies highlight the benefit of oral feedback over written feedback.^35^ Other studies emphasise the importance of having written comments in addition to receiving a numerical score.^36^ Studies have also shown individual feedback to be more effective than group feedback, but owing to time and staffing constraints, it can be more feasible to conduct group feedback.^37^ One of our findings was that through structuring feedback around competencies, trainers and supervisors were able to deliver feedback in various methods. Feedback was delivered to groups based on common weaknesses or to individual trainees and through qualitative or quantitative measures. This structure helped accommodate different preferences of trainees and staffing constraints in addition to retaining feedback specificity.

Competency-based feedback has been implemented in the training of common therapy modalities, such as cognitive–behavioural therapy, to further enhance the efficacy of feedback.^38^ Our findings suggest that EQUIP provides a structure for incorporating a competency-based model into training of these technique-specific skills, including offering key competencies for a range of treatment-specific techniques that can be paired with role-play assessments and used for structuring feedback. EQUIP has already been successfully integrated into multiple mental health interventions, including THP, PM+, Group IPT, TF-CBT and CETA.

Although the above information has succinctly provided practical conclusions, it is also helpful to have a theoretical framework for this study's conclusions. As such, we refer to the ‘mindsponge’ theory, a mechanism to explain how the mind processes new information.^39,40^ The mindsponge theory conceptualises the mind as a sponge containing a set of ‘core values’ unique to each person. New information must effectively exist within the learner's perceivable range. In other words, information must be properly packaged and appeal to the learner's core values.^39^ These two objectives were achieved through competency-based feedback because feedback could be provided in multiple modalities (numerical score, verbal feedback, etc.), allowing for more tailored learning that can better appeal to each learner. Additionally, learners’ core values are often based on their cultures and contexts. Because this study was done in multiple countries and the EQUIP resources were developed with input from a diverse pool of global experts, these resources are likely to be applicable across users from a wide variety of cultural backgrounds and associated core values.

### Challenges to competency-based feedback

Competency-based feedback does not come without challenges. Challenges mentioned in the results section can impede some outcomes of competency-based feedback, such as having actionable feedback if trainees are overwhelmed, correcting harmful behaviour if feedback is not timely, feeling comfortable when giving challenging feedback if cultural norms affect the perception of critical feedback to specific populations, and accommodating the different learning styles of trainees if there are time and scheduling restrictions.

Another challenge was having variability in the depth of responses that participants provided during interviews. In some of our implementation settings, in addition to a didactic learning style being more common, there may not be a strong feedback tradition, and depending on the site and the individual trainee, there was a diversity in feedback experience. Additionally, the ENACT tool used in most settings focuses on common factors and skills, which could further explain some of the more generalised interview responses.

Our study was part of a broader qualitative study for developing the EQUIP platform, and focused only on participants’ perspectives in the competency-based feedback process. Multiple recent EQUIP-related publications have addressed common barriers to training non-specialists in delivering psychological interventions, such as studies showing appropriate reliability (intraclass correlation coefficient of 0.71–0.89) in assessing trainee^41^ success in the implementation of psychological interventions by non-specialists,^24^ and the importance of partnering organisations in supporting the training process.^42^ In addition to these recent studies, we anticipate future studies that will further identify challenges and solutions for EQUIP-supported competency-based training.

### Limitations

There are some pertinent limitations to this study. First, having used a limited set of countries, the results may be limited in generalisability to other countries. Second, the study does not directly investigate whether competency-based feedback is more time-consuming than feedback that does not use a formal structured competency-based approach (although it generally seems well-accepted by participants). Third, this analysis is based on the perceptions of supervisors, trainers and trainees. Although we make arguments about how competency-based feedback might have positive outcomes in terms of learning and skill development, the current study does not explicitly test for this.

### Applications of findings

**Fig. 2:**
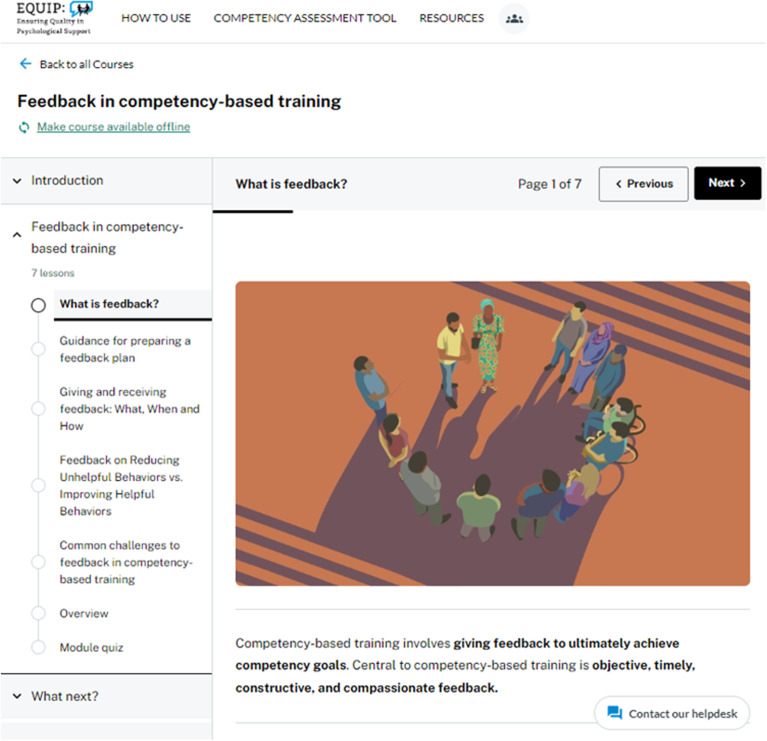
The Ensuring Quality in Psychosocial and Mental Health Care (EQUIP) module on feedback in competency-based training.

Based on our findings, an e-learning module was developed with an iterative, multi-site collaborative approach to support feedback delivery using the EQUIP competency-based approach (Fig. 2). The module addresses the practical elements of giving helpful feedback, which was elucidated in the four key themes of our qualitative findings. The module accomplishes this by examining the following topics: (a) why feedback is essential in competency-based training; (b) knowing the ‘what’, ‘when’ and ‘how’ of providing feedback; (c) preparing a feedback plan with co-trainers, supervisors and trainees that incorporates techniques for supporting the trainees; and (d) managing common challenges that may arise when giving and receiving feedback. The training module contains five lessons in addition to an introduction, overview and quiz with clearly demonstrated visuals, examples and case studies, and was piloted in various EQUIP trainings. These resources on the EQUIP platform allow trainers and supervisors to learn best practices for giving feedback.

In addition to the e-learning module, a series of visualisations were developed for the EQUIP platform. This makes competency assessment results immediately available for trainers and supervisors to share with trainees. The visualisations can be displayed either for individual trainee results for a single assessment or to demonstrate change over time for multiple assessments. Similarly, the visualisations demonstrate group results for a single assessment point as well as change over time for multiple assessments. The visualisations also identify which competencies were most frequently done in a harmful or unhelpful way, and it shows which trainees had the highest number of harmful or unhelpful behaviours so that they can receive extra support from the trainers and supervisors. Moreover, the visualisations for individuals provide the specific behavioural attributes for actions done in an unhelpful way and those done in a helpful manner. This allows trainers and supervisors to provide specific information on both what was done well and what are areas for improvement. This is actionable information that can be given to trainees and is more informative than a single numeric score on a Likert scale competency rating. The immediately available visualizations on the digital platform are consistent with best practices in giving feedback, which requires that learners receive information in a timely and specific manner.

### Future directions

Future research should further investigate the utility of competency-based feedback in psychological intervention training. After the EQUIP programme has more time to implement competency-based feedback, a next step could focus on applying EQUIP's competency-based feedback methods in a prospective study. To date, there is one published study that used EQUIP in competency-based training, which showed an improvement of 18% in competency levels compared with standard training.^43^ More studies will be needed to show the optimal feedback strategies to enhance training through EQUIP. We are interested in understanding whether competency-based feedback actually improves learner outcomes when we have a larger sample size and more time to do exit surveys. This could collate more concrete data on how feedback could be used in different training, supervision or other educational settings. New research could also test the efficacy of feedback loops separately from the confounder of competency-based feedback. Finally, it will be important in the future to link the improved competencies of trainees who learn to deliver psychological interventions via competency-based training methods with improved client outcomes.^44^

In conclusion, this study describes the possible benefits of competency-based feedback to existing non-specialist-led psychological intervention programmes in low- and middle-income countries. Key themes included that competency-based feedback should be personalised and goal-specific; be timely, continuous and bidirectional; be comfortable and objective; and, finally, allow for flexibility in the delivery methods for providing feedback. Future research is needed to evaluate the most effective strategies and methods of training supervisors and trainers to deliver competency-based feedback. Such approaches to training may create a more competent workforce and safer, higher-quality mental healthcare worldwide.

## Supporting information

Elnasseh et al. supplementary material Elnasseh et al. supplementary material

## Data Availability

The data that support the findings of this study are available from the corresponding author, B.A.K., upon reasonable request.
