# Supplementary material for: Perspectives on competency-based feedback for training non-specialists to deliver psychological interventions: multi-site qualitative study of the EQUIP competency-based approach
Source: BJPsych Open. 2024 Jun 3;10(4):e125. doi: 10.1192/bjo.2024.37 (PMC11363075; doi:10.1192/bjo.2024.37)
Supplement: Elnasseh et al. supplementary material [file S2056472424000371sup001.docx]

**Consolidated criteria for reporting qualitative studies (COREQ): 32-item checklist**

Developed from:

Tong A, Sainsbury P, Craig J. Consolidated criteria for reporting qualitative research (COREQ): a 32-item checklist for interviews and focus groups. *International Journal for Quality in Health Care*. 2007. Volume 19, Number 6: pp. 349 – 357

| **No.  Item** | **Criteria description** | **Study Information** |
| --- | --- | --- |
| **Domain 1: Research team and reﬂexivity** |  |  |
| *Personal Characteristics* |  |  |
| 1. Interviewer/ facilitator | Which author/s conducted the interview or focus group? | Ethiopia: One researcher with a PhD and one mental health counseling supervisor with a Master’s level of education  Uganda: One researcher and one monitoring and evaluation personnel both with a Master’s level of education  Peru: Sociologist and qualitative researcher with Master´s level of education.  Lebanon: One research coordinator and one co-investigator both with a Master level of education  Kenya: Two Researchers- A Senior psychiatrist and a Social Worker |
| 2. Credentials | What were the researcher’s credentials? E.g. PhD, MD | Ethiopia: Master and PhD  Uganda: Master, Master  Peru: Master  Lebanon: Master, Master  Kenya: MD, Master, PhD |
| 3. Occupation | What was their occupation at the time of the study? | Ethiopia: Researcher and project focal point at the Center for Victims of Torture  Uganda: Master’s in Public Health and Master’s in Management  Peru: Social research consultant.  Lebanon: Co-investigator of the study and research coordinator  Kenya: Lecturers at the University of Nairobi |
| 4. Gender | Was the researcher male or female? | Ethiopia: One female and one male  Uganda: One female and a male  Peru: Male  Lebanon: One female and one male  Kenya: Both female |
| 5. Experience and training | What experience or training did the researcher have? | Ethiopia: Researcher had 14 years training and experience in research and evaluation, including qualitative methods; focal person has training in counseling and experience providing services in Ethiopia, received qualitative research support through this project  Uganda: Experience in global mental health, economics, research and statistics  Peru: 6+ years of experience in qualitative methods.  Lebanon: One researcher had training in Public Health and research in Health Sciences, with experience in information management in humanitarian sector; one researcher with experience in research of competency assessments for the last 3-4 years before EQUIP in Gaza where they evaluated the process of developing the WEACT competency assessment tools  Kenya: Training in research methods including Qualitative data collection at PhD level and more than 5 years’ experience in research |
| *Relationship with participants* |  |  |
| 6. Relationship established | Was a relationship established prior to study commencement? | Ethiopia: Yes, interviews are with project staff who have ongoing engagement with the interviewers and the project  Uganda: Yes, all interviews were conducted with organization and project staff  Peru: No previous consulting relationship with Socios En Salud.  Lebanon: Yes, research coordinators were involved with supporting some of the facilitators  Kenya: No there was no prior relationship with interviews before study commencement |
| 7. Participant knowledge of the interviewer | What did the participants know about the researcher? e.g., personal goals, reasons for doing the research | Ethiopia: The interview respondents and interviewers knew each other as colleagues  Uganda: The participants were informed as to why the interviews were being conducted. Consent forms were re-read to them before administering the interviews.  Peru: No knowledge except for researcher’s affiliation  Lebanon: Some rater/facilitator knew research members given that they trained them remotely on zoom for several days before.  Kenya: The interviewers were also engaged in training, so they got to know the interviewees well |
| 8. Interviewer characteristics | What characteristics were reported about the interviewer/facilitator? e.g., Bias, assumptions, reasons and interests in the research topic | Ethiopia: Interviewers are known  Uganda: The interviewer was mainly interested in contributing to global mental health through research  Peru: Interest in social topics.  Lebanon: Their educational background and experience and role in the EQUIP study.  Kenya: The interviewers had an academic interest |
| **Domain 2: study design** |  |  |
| *Theoretical framework* |  |  |
| 9. Methodological orientation and Theory | What methodological orientation was stated to underpin the study? e.g. grounded theory, discourse analysis, ethnography, phenomenology, content analysis | The study used a combination of deductive and inductive methodology in a thematic framework analysis approach. |
| *Participant selection* |  |  |
| 10. Sampling | How were participants selected? e.g. purposive, convenience, consecutive, snowball | Ethiopia: Purposive – organizational/project staff  Uganda: Purposive  Peru: Convenience  Lebanon: Purposive- all facilitators were invited for participation in the interviews  Kenya: Purposive- interviews were conducted with participants who were already engaged in the study |
| 11. Method of approach | How were participants approached? e.g. face-to-face, telephone, mail, email | Ethiopia: Face to face, telephone, email  Uganda: Face-to-face  Peru: Telephone and video call.  Lebanon: Face-to-face during training  Kenya: Face-to-face and telephone and email |
| 12. Sample size | How many participants were in the study? | **See Table 1 in the manuscript** |
| 13. Non-participation | How many people refused to participate or dropped out? Reasons? | Ethiopia: None  Uganda: None  Peru: None  Lebanon: 4 facilitators were not available to participate in the focus group discussions.  Kenya: None |
| *Setting* |  |  |
| 14. Setting of data collection | Where was the data collected? e.g., home, clinic, workplace | Ethiopia: Audio call  Uganda: Home, Workplace and training venue  Peru: Community spaces, home and research site.  Lebanon: Video calls  Kenya: Video calls from wherever participants were at that time |
| 15. Presence of non-participants | Was anyone else present besides the participants and researchers? | Ethiopia: No  Uganda: Yes, we had translators  Peru: No  Lebanon: No  Kenya: No |
| 16. Description of sample | What are the important characteristics of the sample? e.g., demographic data, date | Demographic data are included in Table 1. Data collection dates are provided below.  Ethiopia: March to April 2020  Uganda: January to July 2020  Peru: October 2020 to January 2021  Lebanon: November to December 2020  Kenya: February to April 2020 |
| *Data collection* |  |  |
| 17. Interview guide | Were questions, prompts, guides provided by the authors? Was it pilot tested? | Ethiopia: Yes  Uganda: Yes  Peru: Yes  Lebanon: Yes, piloted by other sites not  Kenya: Yes |
| 18. Repeat interviews | Were repeat interviews carried out? If yes, how many? | Ethiopia: No  Uganda: No  Peru: No  Lebanon: No  Kenya: No |
| 19. Audio/visual recording | Did the research use audio or visual recording to collect the data? | Ethiopia: Audio  Uganda: Audio/video recording  Peru: Audio recording.  Lebanon: Audio/video recording  Kenya: Audio/Video recording |
| 20. Field notes | Were ﬁeld notes made during and/or after the interview or focus group? | Ethiopia: Yes  Uganda: Yes  Peru: No  Lebanon: Yes  Kenya: No |
| 21. Duration | What was the duration of the interviews or focus group? | Ethiopia: 35-75 min  Uganda: 25-50 mins  Peru: 40 – 60 minutes  Lebanon: 60 – 120 minutes  Kenya: 45 – 60 minutes |
| 22. Data saturation | Was data saturation discussed? | As study samples in some sites were small, it was not possible to aim for or examine data saturation at the site level or for minor themes. For the presence of major themes, data saturation was achieved in the total/cross-country sample. |
| 23. Transcripts returned | Were transcripts returned to participants for comment and/or correction? | Ethiopia: No  Uganda: No  Peru: No  Lebanon: No  Kenya: No |
| **Domain 3: analysis and ﬁndings** |  |  |
| *Data analysis* |  |  |
| 24. Number of data coders | How many data coders coded the data? | Four persons were involved in coding and summarizing all data received from the sites. |
| 25. Description of the coding tree | Did authors provide a description of the coding tree? | A thematic coding framework was created to structure multiple-researcher coding. Inductive coding was used to identify additional codes. The coding framework was charted into tables to compare various stakeholders’ experiences and views. Then, relevant subthemes evident in the data were identified. Cross-country findings on each theme were summarized for interviews/focus groups with supervisors, trainers, and trainees who participated in various psychological intervention trainings specific to each site (e.g., Ethiopia using Problem Management Plus training, Peru using Thinking Healthy Program training) with a particular focus on their experiences with giving and receiving feedback structured around competency-based assessments during training and supervision. |
| 26. Derivation of themes | Were themes identiﬁed in advance or derived from the data? | Both. Pre-developed thematic coding was initially used, and inductive coding was then used to identify additional codes. |
| 27. Software | What software, if applicable, was used to manage the data? | Results from interviews/focus group discussion were transferred into Dedoose software to code the data and for cross-site analysis. |
| 28. Participant checking | Did participants provide feedback on the ﬁndings? | Participants did not provide feedback on the findings. |
| *Reporting* |  |  |
| 29. Quotations presented | Were participant quotations presented to illustrate the themes/ﬁndings? Was each quotation identiﬁed? e.g. participant number | Quotations were presented to illustrate major themes and findings. Basic demographic description (participant type, country) was given for the person making each of these statements. Participant numbers were used within sites but not pooled across sites for the cross-country write up. |
| 30. Data and ﬁndings consistent | Was there consistency between the data presented and the ﬁndings? | Yes |
| 31. Clarity of major themes | Were major themes clearly presented in the ﬁndings? | Yes (major headings in the results section) |
| 32. Clarity of minor themes | Is there a description of diverse cases or discussion of minor themes? | Yes (sub-headings in the results section) |
